# Nucleoside Analogue with Thymidine Nucleobase Inhibits *Leishmania infantum* and Depolarizes the Plasma Membrane Potential In Vitro

**DOI:** 10.1021/acsomega.5c09199

**Published:** 2026-02-26

**Authors:** Clarissa Menezes, Ingrid de O. Dias, Elisa Pileggi, Andre G. Tempone, Fabrizio Pertusati, Samanta E. T. Borborema

**Affiliations:** † Center for Parasitology and Mycology, 89119 Adolfo Lutz Institute, Sao Paulo 01246-000, Brazil; ‡ Post-Graduation Program of the Diseases Control Coordination, Secretary of State of Health of Sao Paulo, Sao Paulo 01246-900, Brazil; § School of Chemistry, Cardiff University, Cardiff CF10 3AT, Wales, United Kingdom; ∥ Physiopathology Laboratory, 196591 Butantan Institute, Sao Paulo 05503-900, Brazil

## Abstract

Visceral leishmaniasis, caused by the protozoan parasites *Leishmania infantum* or *L. donovani*, remains a lethal neglected tropical disease without effective therapy. Current drugs available are toxic, leading to severe side effects, treatment abandonment, and resistance, underscoring the urgent need for new therapeutic options. As *Leishmania* spp*.* are auxotrophic for purines, they rely on the purine salvage pathway for their nucleotide biosynthesis. Consequently, nucleoside analogues (NAs) represent a promising class of compounds for the design of antiparasitic agents. This study aimed to investigate the in vitro antileishmanial activity of NA compounds and the cellular alterations induced by treatment. Twelve NA compounds were screened for antileishmanial activity against promastigote and amastigote forms of *L. infantum* and cytotoxic effects in mammalian cells. Among all compounds evaluated, six of them demonstrated antipromastigote activity, exhibiting 50% effective concentration (EC_50_) values ranging from 9.29 to 76.74 μM. As for amastigote activity, two compounds, **5** and **10**, were effective, with values of EC_50_ of 8.02 and 31.95 μM, respectively. Only compound **1** maintained cellular viability at the maximum concentration tested (>200 μM). The selective index for the derivatives investigated ranges from 0.5 to 4.2. Compound **5**, a tritylated thymidine NA, the most active, was further subjected to analysis of cellular alterations using fluorescent-based approaches. This analogue demonstrated a lack of cytotoxicity against murine peritoneal macrophages up to 50 μM and nonhemolytic activity up to 100 μM. When applied at EC_50_, it did not cause damage to plasma membrane permeability, the integrity of the genetic material, acidocalcisomes, intracellular Ca^2+^, and ROS levels of treated *Leishmania* parasites. However, it caused depolarization of the plasma membrane potential, leading to cell death. Further studies are also necessary to understand the enzymatic action of this most active compound, and optimization is required to develop more effective and safer antileishmanial lead compounds. In conclusion, compound **5** might be a suitable candidate for the development of antileishmanial agents.

## Graphical abstract

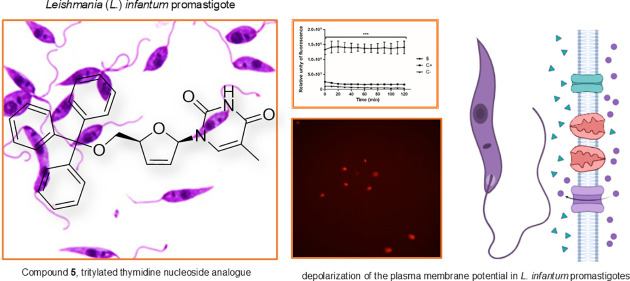

## 1 Introduction

Leishmaniases are neglected tropical diseases with devastating human, social, and economic impacts worldwide, particularly in tropical and subtropical regions. These diseases disproportionately affect socioeconomically disadvantaged and marginalized populations; moreover, there exist gaps in the strategic plans for prevention, control, elimination, and eradication.^1^ They are vector-borne infectious parasitic infections caused by the protozoa of the genus *Leishmania* and are transmitted to mammalian hosts through the bite of infected female phlebotomine sandflies. Clinical manifestations vary significantly in severity, ranging from self-limiting cutaneous lesions to potentially fatal systemic visceral involvement, unless treated. An estimated 0.7–1 million new cases are reported per year from nearly 100 endemic countries. Visceral leishmaniasis is caused by *Leishmania (Leishmania) donovani* in Asia and Africa and *Leishmania (L.) infantum* in the Mediterranean Basin, the Middle East, Central Asia, South America, and Central America.^2^ Due to the lack of a vaccine and the serious adverse effects caused by the existing drugs, there is a critical need for the development of new antileishmanial agents and designing drug candidates with distinct chemical scaffolds and mechanisms of action compared to current therapeutics.

Unlike their mammalian and insect hosts, *Leishmania* parasites lack the biosynthetic machinery for de novo purine nucleotide synthesis. Consequently, they are auxotrophic for purines, relying exclusively on the salvage pathway to meet their metabolic requirements. Within this framework, nucleoside transporters are essential for the translocation of exogenous purine bases across the parasite’s plasma membrane. This obligatory dependence on purine salvage presents a compelling paradigm for drug targeting.^3,4^ Though pyrimidine is synthesized by both de novo and through the pyrimidine salvage pathway, enzymes as well as the nucleoside analogues (NA) that may interfere with these pathways constitute a promising source for novel treatments against *Leishmania.* ^5–7^

NA are synthetic compounds structurally similar to natural nucleosides and have been extensively studied for their antiviral and antimicrobial properties.^8–10^ Many purine and pyrimidine analogues with anti-*Leishmania* activities have been explored (Nwoke et al.^7^). However, allopurinol is the only NA drug in the clinic against leishmaniasis.^11^

The NA molecules do not cross biological membranes very well due to their high polarity. A common strategy to overcome this drawback is to transform these molecules into prodrugs^10,12^ or functionalize them with lipophilic functional groups like triphenylmethyl (trityl),^13^ then make the NA more permeable through the cellular membrane.

Based on this, we develop NA compounds to treat flavivirus^14^ and other viral infections.^15^ Hampton and colleagues extended this approach to parasitic diseases by preparing a series of uridine derivatives as inhibitors of deoxyuridine 5′-triphosphate nucleotidohydrolase (dUTPase), an essential enzyme in nucleoside metabolism.^16^ A variety of analogues of dUMP were described where the substituents were introduced at the 3′- and 5′-positions, together with variation in the heteroatom at the 5′-position. Ruda and colleagues explored the effects of the substituted trityl group, its position in the deoxyU, and chemical variation of the nucleobase, as inhibitors of dUTPase of *Plasmodium falciparum*, responsible for malaria disease.^17^

This prompted us to prepare a series of tritylated nucleoside analogues (Figure 1) that would also include purine nucleobases to expand the applicability of this strategy to act as antiparasitic agents. We included tritylated acyclic nucleoside analogues (ANAs), those in which the sugar moiety is replaced by a tritylated aliphatic chain. ANAs have been at the cornerstone of antiviral treatment,^10^ and their use as antiparasitic potential drugs has been raised.^18,19^ Recently, these molecules have found application in the treatment of protozoan diseases such as trypanosomiasis, babesiosis, Chagas disease, and leishmaniasis. Particularly attractive are acyclic nucleoside phosphonates (ANPs) that can inhibit phosphoribosyl transferases (PRTs), the key enzymes of the purine salvage pathway.^20^

**Figure 1:**
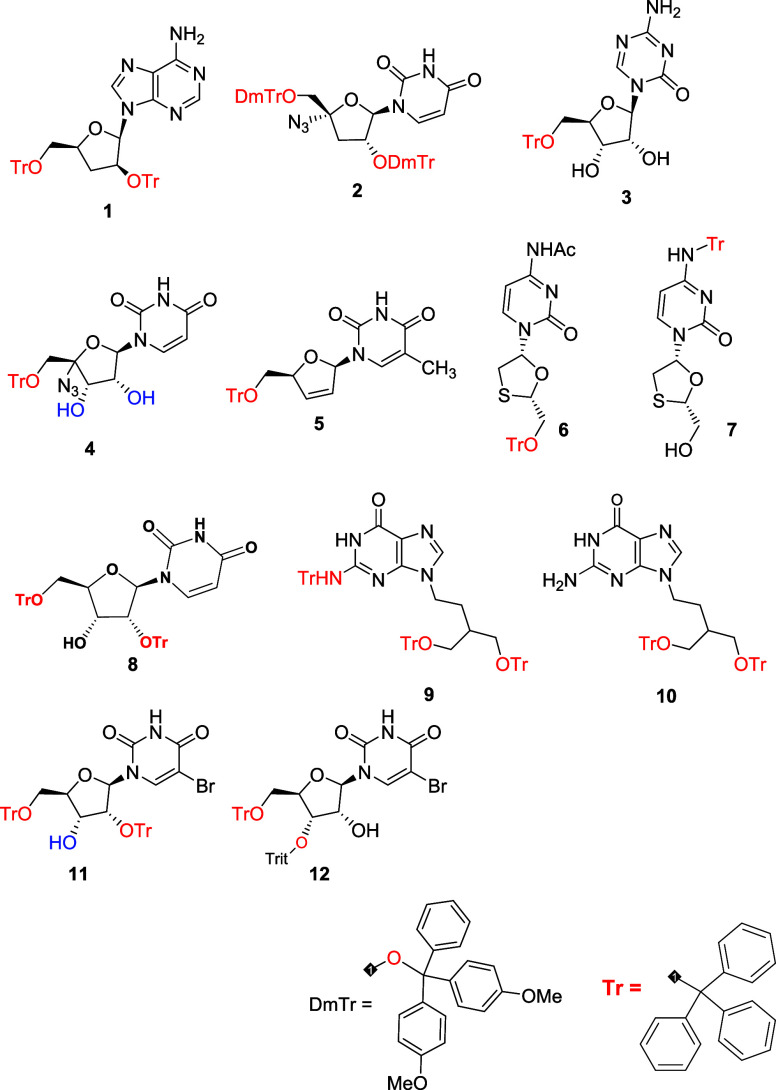
Chemical structure of nucleoside analogs.

In this article, we report the investigation of a series of 12 tritylated NAs where modification of the nucleobase ring, as well as tritylation at different positions in the sugar moiety, is effective against *L. infantum*. Of previously reported structures, we have identified tritylated penciclovir^13^ and stavudine as hit compounds against both forms of *Leishmania* parasite in the micromolar range; the most active was further investigated for its mechanism of action. Our results demonstrate that other biological targets other than dUTPase may be the target of our molecules.

## 2 Results

### 2.1 In Vitro Activity against *L. infantum* and Mammalian Cytotoxicity

Cell-based assays were performed to evaluate the effective activities of all 12 synthesized compounds in extracellular promastigotes and intracellular amastigotes of *L. infantum* and mammalian NCTC cells following treatment (Table 1).

**Table 1:** Antileishmanial Activity, Cytotoxicity, and Selectivity Index for Nucleoside Analogue Compounds^a^

**compound**	**EC**_**50**_ **promastigote**	**EC**_**50**_ **amastigote**	**CC**_**50**_ **NCTC**	**CC**_**50**_ **macrophage**	**SI** ^b^	**SI** ^c^
**1**	>150	>50	>200	ND	ND	ND
**2**	>150	>50	12.67 ± 0.86	ND	ND	ND
**3**	>150	>50	8.98 ± 1.82	ND	ND	ND
**4**	9.29 ± 0.53	>50	1.80 ± 0.39	ND	ND	ND
**5**	27.67 ± 0.64	8.02 ± 3.16	3.84 ± 0.51	>50	>6.2	0.5
**6**	13.27 ± 1.59	>50	4.46 ± 1.34	ND	ND	ND
**7**	25.36 ± 4.74	>50	25.52 ± 4.11	ND	ND	ND
**8**	>150	>50	137.75 ± 16.75	ND	ND	ND
**9**	>150	>50	147.50 ± 2.50	ND	ND	ND
**10**	76.74 ± 5.43	31.95 ± 2.75	133.15 ± 6.25	ND	ND	4.2
**11**	47.81 ± 4.22	>50	8.04 ± 0.67	ND	ND	ND
**12**	>150	>50	4.78 ± 0.16	ND	ND	ND
miltefosine	16.69 ± 3.49	17.80 ± 1.39	116.70 ± 5.30	ND	ND	6.5

a EC_50_: 50% effective concentration and CC_50_: 50% cytotoxic concentration are expressed in μM. SI = selectivity index was calculated by dividing the CC_50_ by the EC_50_ against the amastigote.

b SI related to CC_50_ in macrophage,

c SI related to CC_50_ in NCTC. ND = not determined. The results are expressed as mean ± SEM of three independent experiments, which were performed in duplicate.

The activity against promastigotes was evaluated using the colorimetric MTT reduction assay to assess mitochondrial metabolic function. Among 12 compounds evaluated, six of them demonstrated antipromastigote activity, exhibiting EC_50_ values ranging from 9.29 to 76.74 μM.

The antileishmanial activity against intracellular amastigotes was determined using quantitative light microscopy by counting Giemsa-stained infected macrophages. Two compounds, **5** and **10,** were effective with values of EC_50_ of 8.02 and 31.95 μM, respectively.

Cytotoxicity assays were performed against the NCTC cell line using the colorimetric MTT method. Compound **1** demonstrated a lack of cytotoxic effects even at the maximum concentration tested (>200 μM), and the other 11 showed cytotoxicity ranging from 1.80 to 147.50 μM. The SI related to amastigotes ranges from 0.5 to 4.2.

Compound **5** showed activity against both forms of the parasite and was the most active against the amastigote (Figure 2). As presented in Figure 3, treatment with compound **5** (25 μM) eliminated more than 90% of the intracellular amastigotes, and at 6.25 μM eliminated approximately 50% of the parasite and maintained the macrophage morphology. Then it was subjected to the mechanism of action (MoA) assays to analyze cellular alterations.

**Figure 2:**
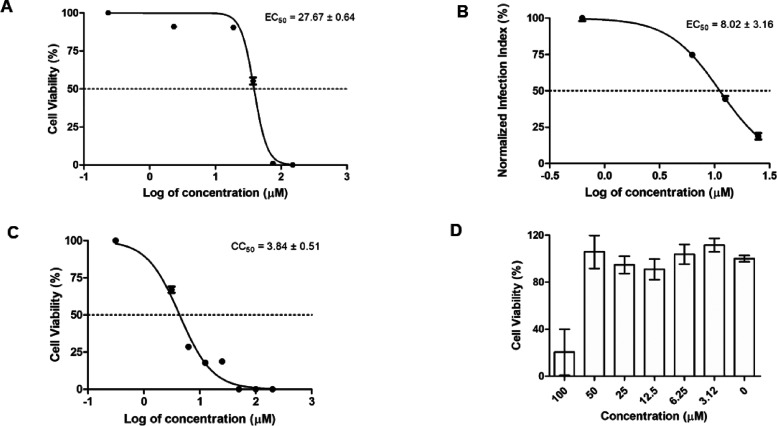
Determination of the anti-*L. infantum* activity and cytotoxicity of the compound **5**. (A) 50% effective concentration (EC_50_) against promastigotes treated for 48 h and viability of cells was determined by the MTT assay; (B) 50% effective concentration (EC_50_) against intracellular amastigotes treated for 96 h and infection index was determined by microscopic counting; (C) 50% cytotoxic concentration (CC_50_) against NCTC cells treated for 48 h and viability of cells was determined by the MTT assay; (D) cytotoxicity against peritoneal macrophages treated for 48 h and viability determined by the MTT assay. The results are expressed as mean ± SEM of three independent experiments, which were performed in duplicate.

**Figure 3:**
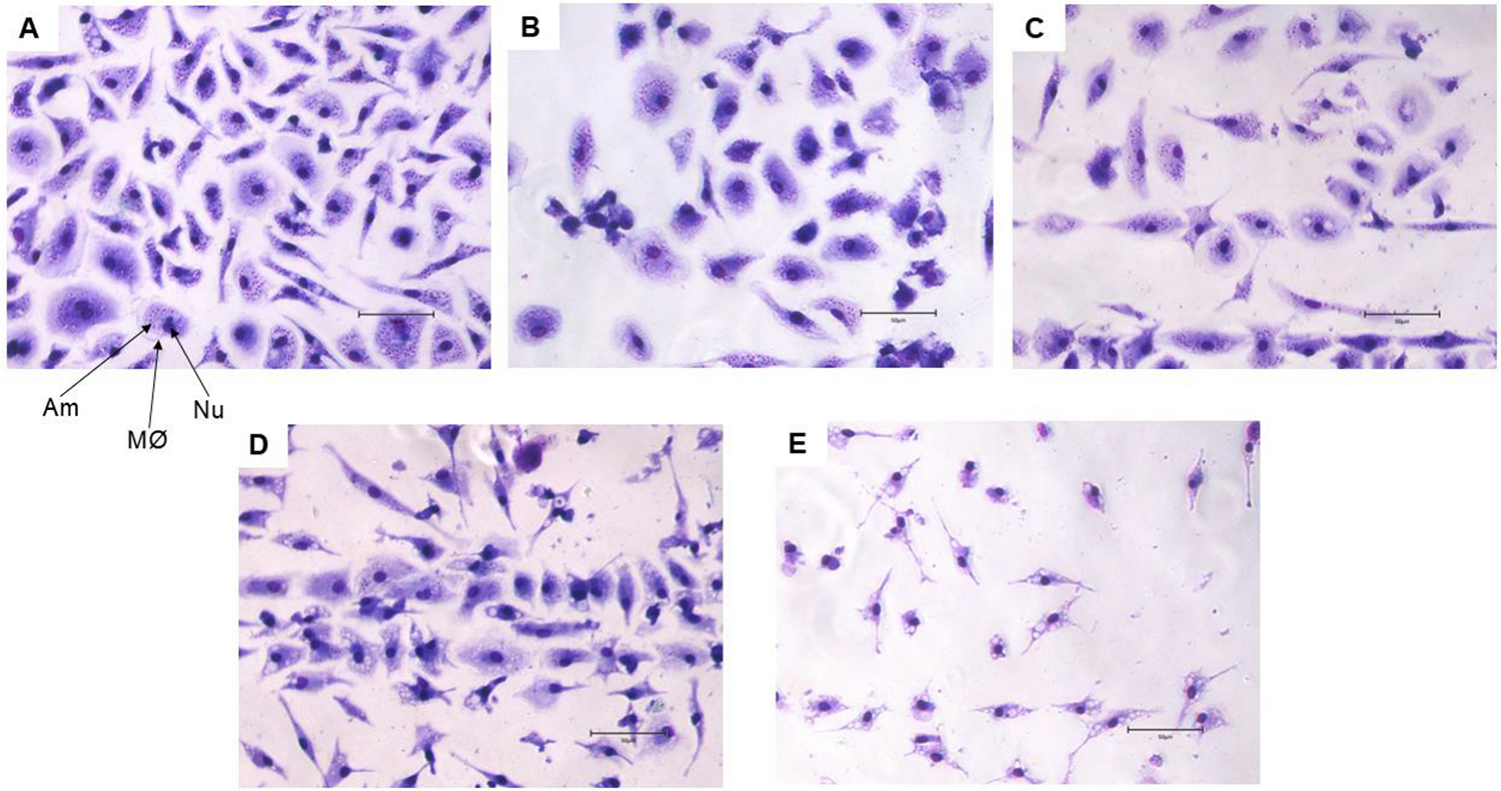
Micrography by optical microscopy of *L. infantum* amastigotes-infected murine macrophage treated with compound **5** for 96 h. (A) Untreated infected macrophage; (B) infected macrophages treated with 6.25 μM; (C) infected macrophages treated with 12.5 μM; (D) infected macrophages treated with 25 μM; (E) infected macrophages treated with 50 μM. MØ = macrophage; Nu = nucleus of macrophage; Am = intracellular amastigote. The right bottom bar represents 50 μm. Objectives of 40×.

### 2.2 Effect of Compounds on Murine Cell Lines

The effect of compound **5** on murine erythrocytes was evaluated by spectrophotometric analysis by measuring the lysis of red blood cells. No significant lysis of erythrocytes was observed up to 100 μM. Incubation with bidistilled water was employed as a positive control, resulting in 100% hemolysis. To evaluate the cytotoxic profile of the potent compound **5** on murine macrophages, an MTT assay was performed; no significant reduction in cell viability was observed up to the maximum concentration of 50 μM. The SI related to amastigotes was >6.2 (Figure 2D).

### 2.3 In Vitro Mechanism of Action on *L. infantum* Promastigote

A short-term dose–response assay was performed to determine the EC_50_ of compound **5**. As the EC_50_ was calculated to be 55 μM at 2 h (Figure 4), all subsequent experiments were performed using this standardized concentration.

**Figure 4:**
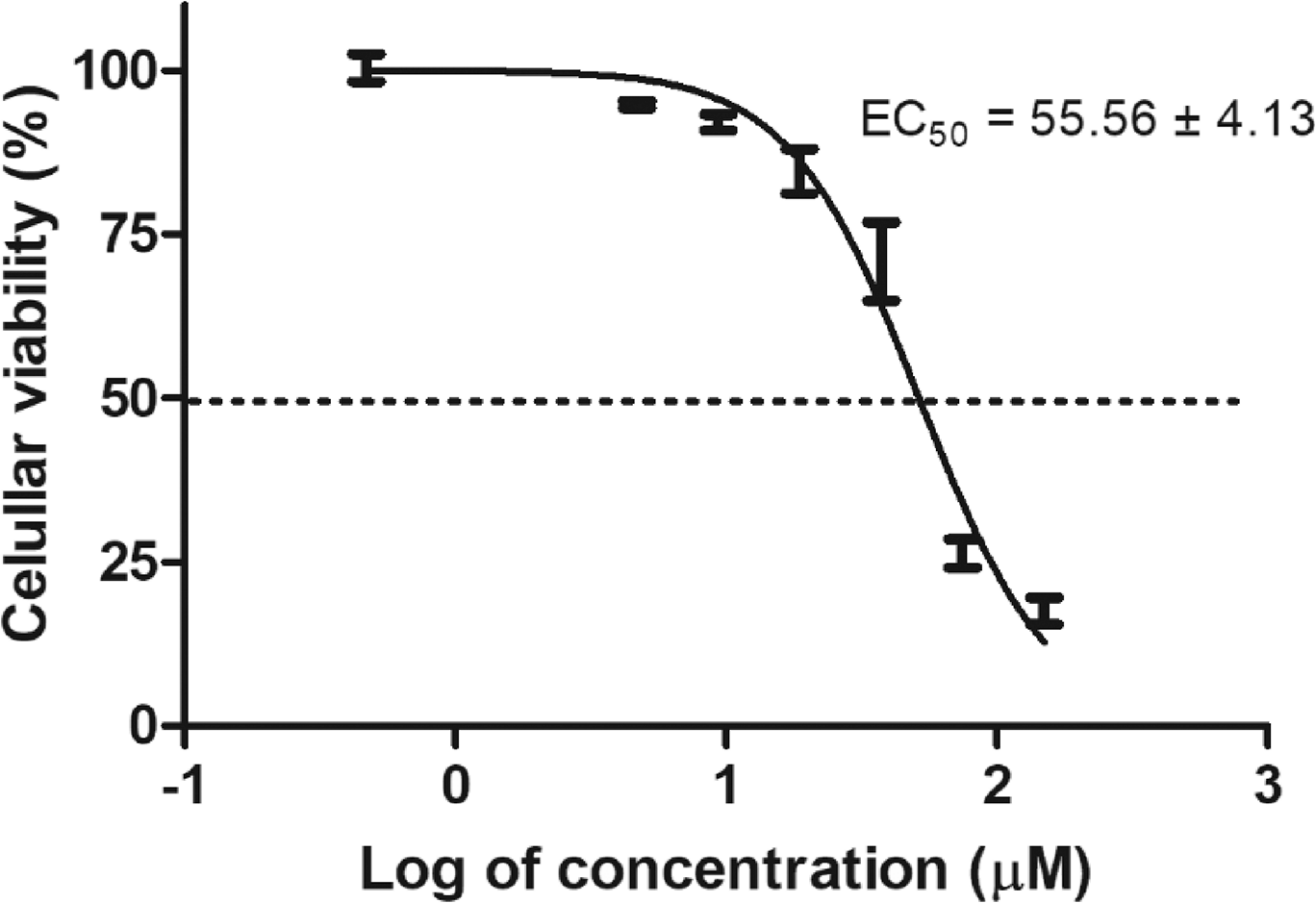
Determination of the 50% effective concentration (EC_50_) for compound **5**. Promastigotes (2 × 10^6^/well) were treated for 2 h. Viability was subsequently assessed by MTT reduction assay.

### 2.4 Plasma Membrane Integrity

The plasma membrane permeability was evaluated by spectrofluorometric analysis and fluorescence microscopy using the fluorescent probe SYTOX Green. As presented in Figure 5A, treatment with compound **5** showed no alteration in the plasma membrane permeability of *Leishmania*, with fluorescence levels like untreated parasites, even after 2 h of incubation. Parasites treated with compound **5** presented few fluorescent cell structures (Figure 5B–E). Triton X-100 was used as a positive control and showed higher fluorescence levels, promoting significant permeabilization of the plasma membrane.

**Figure 5:**
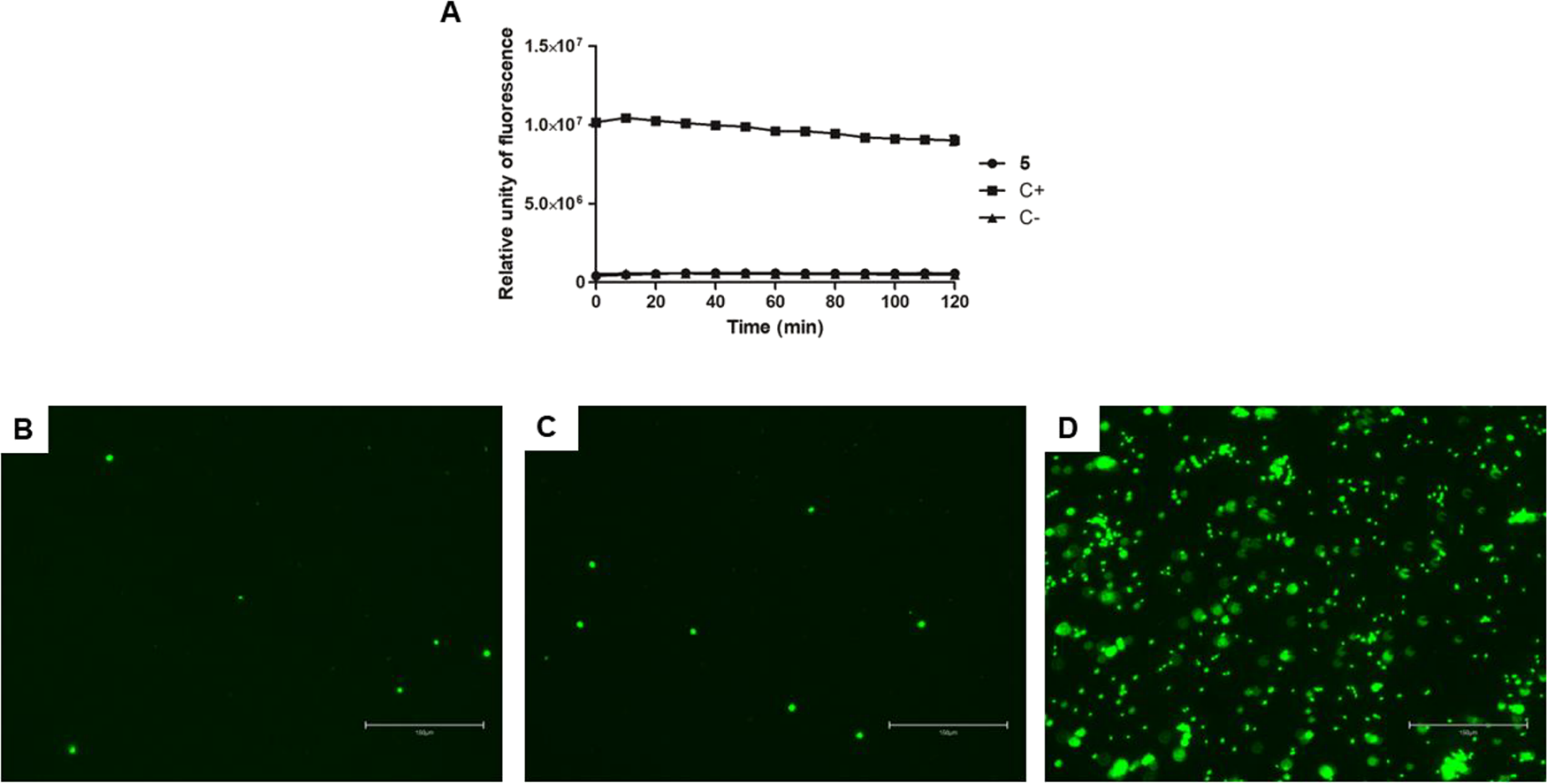
Evaluation of plasma membrane permeability in *L. infantum* promastigotes treated with compound **5** (55 μM) for 2 h using fluorescent probe SYTOX Green. (A) Spectrofluorometer measurement; (B) digital fluorescent microscopy of the untreated parasite, negative control; (C) digital fluorescent microscopy of the treated parasite with compound **5;** (D) digital fluorescent microscopy of the treated parasite with Triton X-100 (0.5%), positive control. The right bottom bar represents 150 μm. Objectives of 20×.

The plasma membrane electric potential was evaluated by spectrofluorometric analysis and fluorescence microscopy using the fluorescent probe DiSBAC_2_(3). As presented in Figure 6A, treatment with compound **5** showed a significant alteration (*p* < 0.05) of the membrane electric potential at the first time of incubation with an increase in fluorescence levels when compared to the untreated parasites. Miltefosine was used as a positive control, promoting a slight alteration.

**Figure 6:**
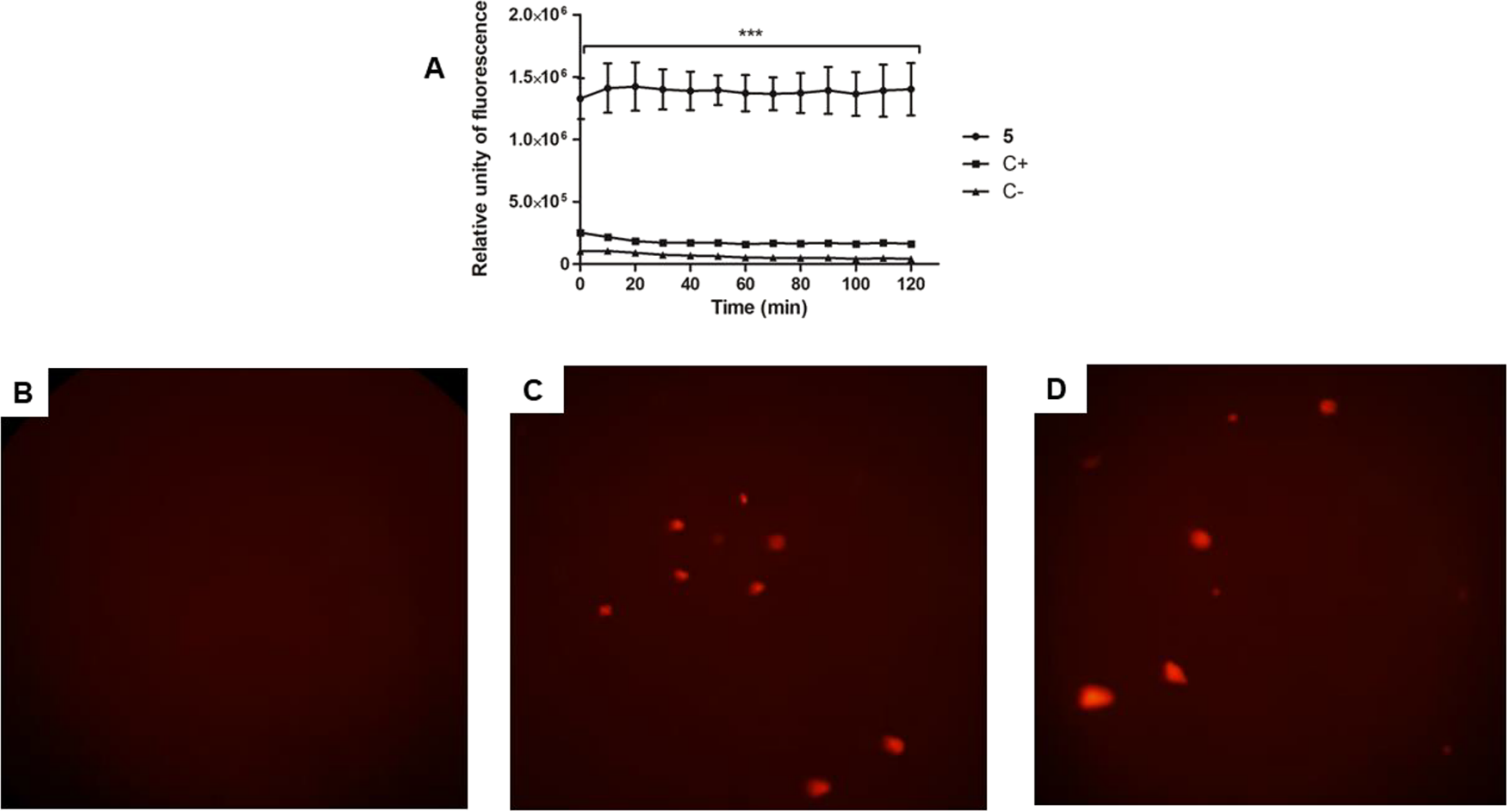
Evaluation of plasma membrane electric potential in *L. infantum* promastigotes treated with compound **5** (55 μM) for 2 h using the fluorescent probe DiSBAC_2_(3). (A) Spectrofluorometer measurement; (B) digital fluorescent microscopy of the untreated parasite, negative control; (C) digital fluorescent microscopy of the treated parasite with compound **5;** (D) digital fluorescent microscopy of the treated parasite with miltefosine (35 μM), positive control. Objectives of 40×.

Using digital fluorescence microscopy, it was also possible to corroborate the spectrofluorimetric data by taking images of the parasites after incubation with compound **5**. Treated parasites presented fluorescent cell structures like positive control cells, corresponding to depolarization (Figure 6B–E).

### 2.5 DNA Integrity

The DNA content of promastigotes was investigated after treatment with compound **5**. As shown in Figure 7A, no alteration in the integrity of the genetic content was observed, with a band of similar intensity and characteristics to the untreated parasites. The positive control, H_2_O_2_, exhibited a lower intensity band, illustrating DNA fragmentation.

**Figure 7:**
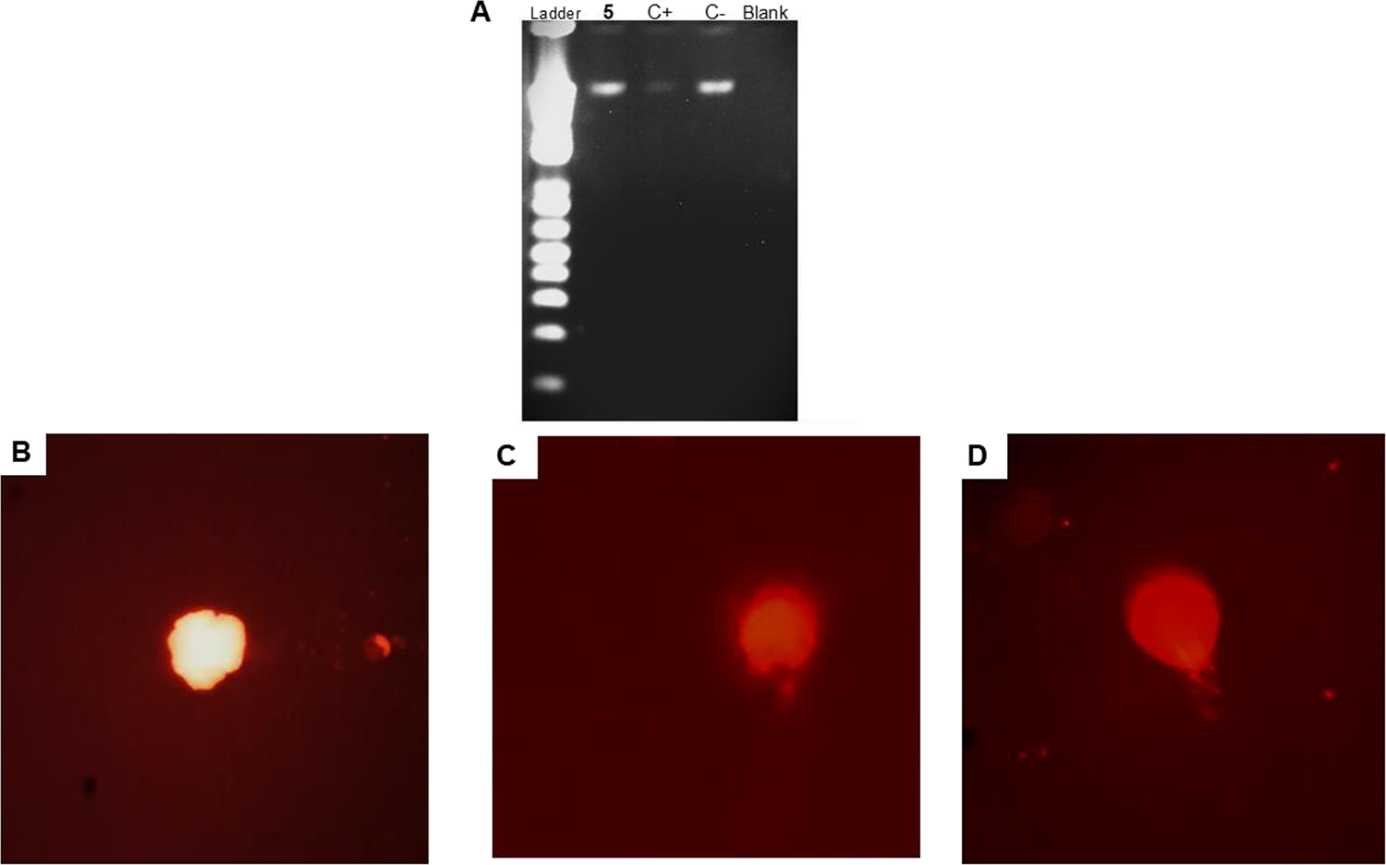
Evaluation of DNA integrity in *L. infantum* promastigotes treated with compound **5** (55 μM) for 2 h. (A) 2% agarose gel electrophoresis stained with ethidium bromide (0.5 μg/mL). A ladder of 1 Kb was used as a standard; (B) fluorescent microscopy of the untreated parasite, negative control; (C) fluorescent microscopy of the treated parasite with compound **5;** (D) fluorescent microscopy of the treated parasite with H_2_O_2_ (10 mM), positive control. Objectives of 40×.

As determined by conventional comet assays, *L. infantum* treated with compound **5** presents slight DNA damage and the formation of a halo around the nucleus (head), but no tail. The positive control exhibited a gradual increase in the length and intensity of the comet tail, corresponding to cells with a significant number of DNA strand breaks (Figure 7B–D).

### 2.6 Reactive Oxygen Species

The reactive oxygen species (ROS) content was investigated by spectrofluorometric analysis and fluorescence microscopy using the fluorescent probe H_2_DCFDA. According to the data (Figure 8A), compound **5** did not alter the ROS levels when compared to untreated parasites. The treated parasites did not present fluorescent cell structures (Figure 8B–E). In contrast, the parasite treated with sodium azide, the positive control, showed higher fluorescence levels, increasing the ROS production.

**Figure 8:**
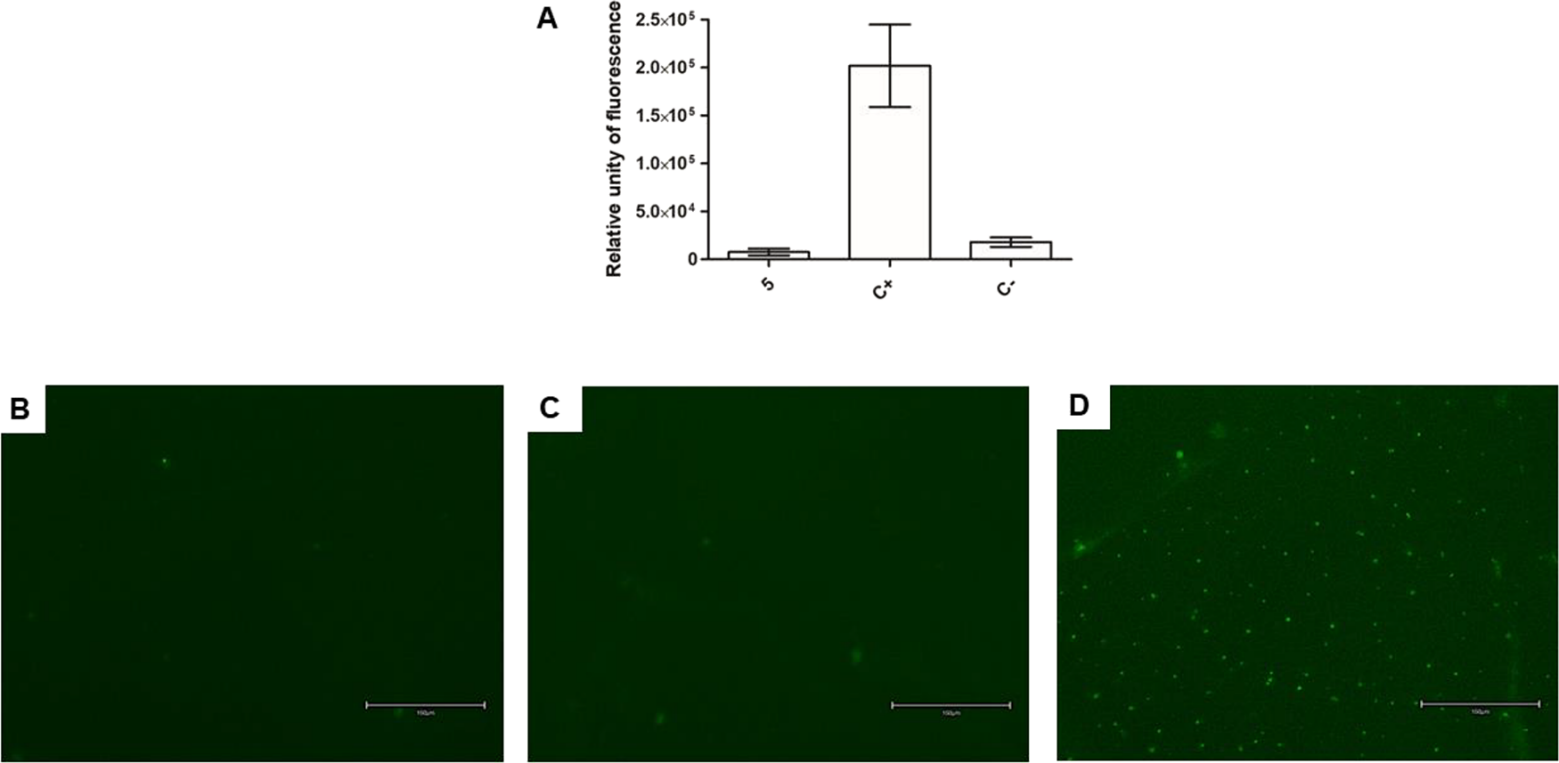
Evaluation of reactive oxygen species levels in *L. infantum* promastigotes treated with compound **5** (55 μM) for 2 h using the fluorescent probe H_2_DCFDA. (A) Spectrofluorometer measurement; (B) fluorescent microscopy of the untreated parasite, negative control; (C) fluorescent microscopy of the treated parasite with compound **5;** (D) fluorescent microscopy of the treated parasite with sodium azide (10 mM), positive control. The right bottom bar represents 150 μm. Objectives of 20×.

### 2.7 Intracellular Calcium Ions (Ca^2+^)

The intracellular content of Ca^2+^ was investigated by spectrofluorometric analysis and fluorescence microscopy using the fluorescent probe Fluo-4 AM. According to the data (Figure 9A), compound **5** did not alter the Ca^2+^ levels when compared to untreated parasites. The treated parasite did not present fluorescent cell structures (Figure 9B–D). In contrast, the parasite treated with Triton X-100, the positive control, showed higher fluorescence levels, increasing the content of Ca^2+^.

**Figure 9:**
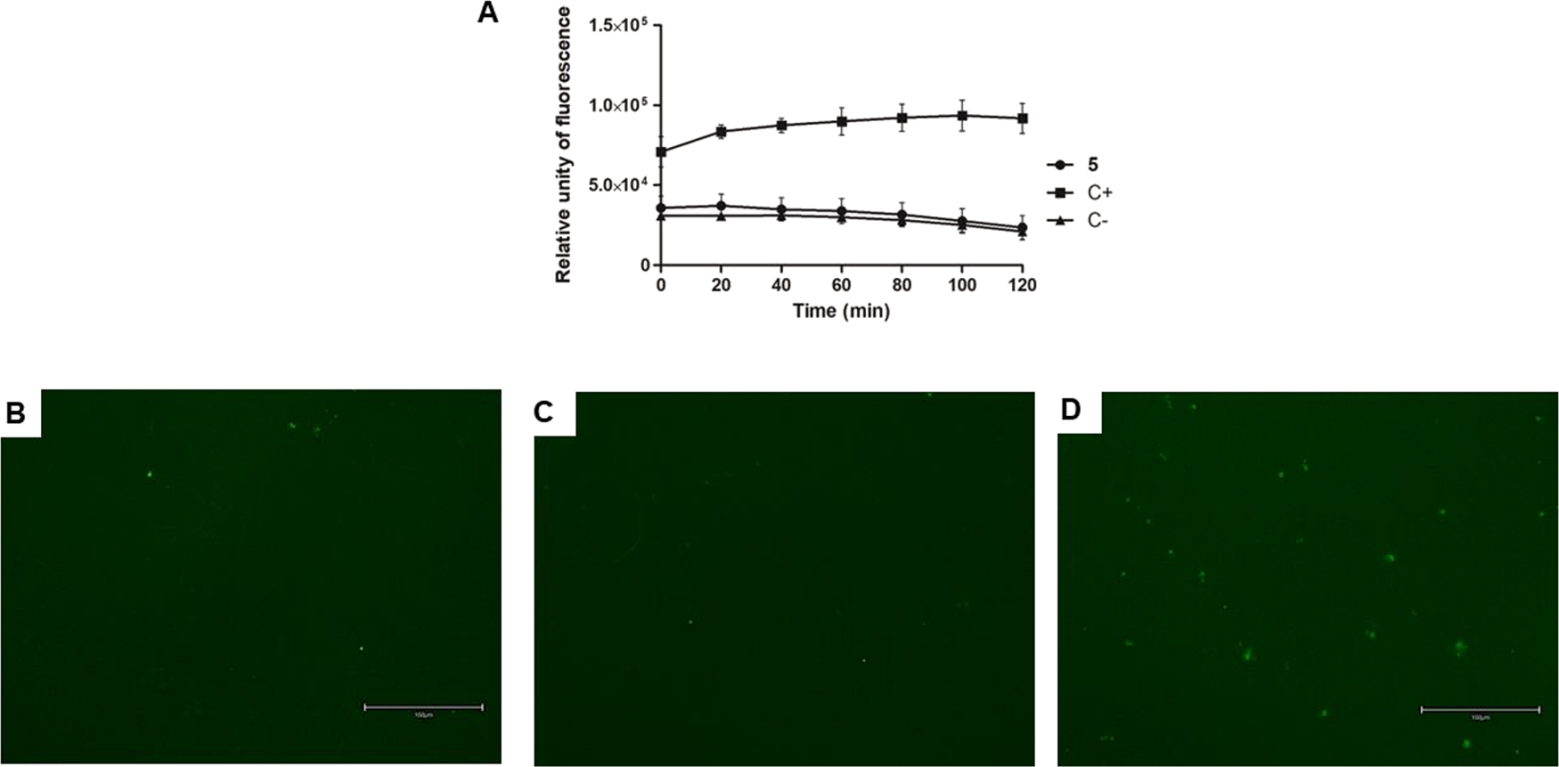
Evaluation of Ca^2+^ levels in *L. infantum* promastigotes treated with compound **5** (55 μM) for 2 h using the fluorescent probe Fluo-4 AM. (A) Spectrofluorometer measurement; (B) digital fluorescent microscopy of the untreated parasite, negative control; (C) digital fluorescent microscopy of the treated parasite with compound **5**; (D) digital fluorescent microscopy of the treated parasite with Triton X-100 (0.5%), positive control. The right bottom bar represents 150 μm. Objectives of 20×.

### 2.8 Acidocalcisomes

The acidocalcisomes were investigated by spectrofluorometric analysis and fluorescence microscopy using the fluorescent probe acridine orange. As demonstrated in Figure 10A, compound **5** did not interfere with this organelle when compared to untreated parasites. The treated parasite did not present fluorescent cell structures (Figure 10B–D). In contrast, the parasite treated with nigericin, the positive control, showed higher fluorescence levels, altering the content of acidocalcisomes.

**Figure 10:**
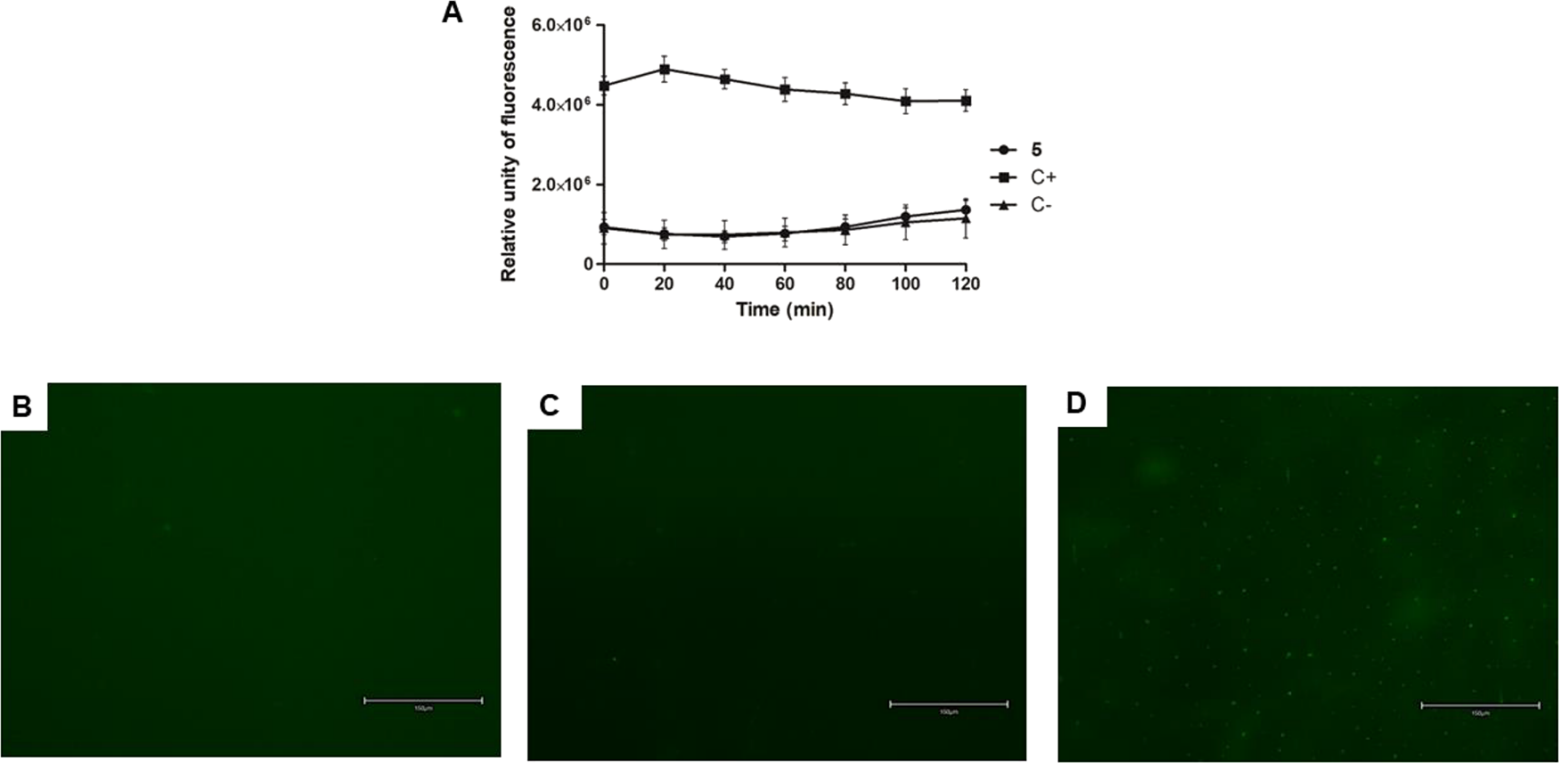
Evaluation of acidocalcisome in *L. infantum* promastigotes treated with compound **5** (55 μM) for 2 h using the fluorescent probe acridine orange. (A) Spectrofluorometer measurement; (B) digital fluorescent microscopy of the untreated parasite, negative control; (C) digital fluorescent microscopy of the treated parasite with compound **5**; (D) digital fluorescent microscopy of the treated parasite with nigericin (4 μM), positive control. The right bottom bar represents 150 μm. Objectives of 20×.

## 3 Discussion

In the search for new drug hits for VL, we are reporting the investigation of a series of nucleoside analogue compounds against *L. infantum*. *Leishmania* is unable to synthesize purines endogenously and must salvage preformed purines from its host environment to satisfy metabolic requirements. Though pyrimidine is synthesized by both de novo and through the pyrimidine salvage pathway, targeting these pathways using nucleoside analogues represents a promising therapeutic strategy to disrupt parasite metabolism and develop novel treatments for leishmaniasis.^7^ Allopurinol is the only thymidine purine analogue drug used clinically to treat leishmaniasis. Also in veterinary medicine, it is the main drug used for the treatment of canine leishmaniasis, in single or combination therapy.^11^ It is converted intracellularly into allopurinol ribonucleoside, and the mechanism of action is related to the disruption of RNA synthesis, blocking protein synthesis, and consequently selective parasite death.^21^

The clinical efficacy of nucleoside analogues is frequently hindered by suboptimal pharmacokinetic properties. However, the development of focused chemical libraries through strategic structural modifications can overcome these limitations. Furthermore, the implementation of prodrug strategies represents a viable approach to improve systemic bioavailability and phosphorylation efficiency of nucleoside analogues^10,12^

Drug repurposing has emerged as a highly effective strategy for accelerating the drug discovery and development pipeline by identifying new therapeutic applications for existing drugs. This approach significantly mitigates the risk of clinical failure, reduces developmental costs and time to market.^22^ All drugs active against leishmaniasis have arisen via repurposing, including antimonials, amphotericin B, miltefosine, paromomycin, and pentamidine.^23,24^ Similarly, nucleoside drugs currently in use to treat other manifestations could be investigated for potential repurposing for leishmaniasis.

Following a phenotypic screening campaign conducted by GlaxoSmithKline,^25^ a series of pyrrolopyrimidines, nucleoside derivatives, was optimized, and DNDI-6174 was identified as a promising oral preclinical candidate for the treatment of leishmaniasis. DNDI-6174 showed potent in vitro antileishmanial activity in submicromolar concentrations against a range of *Leishmania* species, drug-resistant strains, and clinical isolates. This pyrrolopyrimidine inhibits *Leishmania* cytochrome bc1 complex (III) of the parasite’s electron transport chain and interacts with the Qi active site of this mitochondrial enzyme.^26^

In our study, six of 12 nucleoside analogue compounds previously investigated as antimicrobials^13,9^ also showed potent antileishmanial activity in micromolar concentrations. The studies with the intracellular amastigotes revealed a potent activity of the compound **5** against the clinically relevant form of the parasite (EC_50_ = 8.0 μM), being two times more active than miltefosine (EC_50_ = 17.8 μM), the oral drug used to treat VL caused by *L. donovani*.

The antileishmanial effect of nucleoside analogues based on the inhibition of the purine metabolism pathway is well established.^7,21^ We also showed that the compound **10**, an adenosine analogue compound, tritylated penciclovir, was active against both forms of the parasite. Among the novel analogues evaluated in this study, several pyrimidine nucleoside analogues (**4**, **5**, **6**, **7,** and **11**) exhibited significant antiprotozoal activity against *L. infantum* promastigotes. To our knowledge, our study has demonstrated the antileishmanial activity of these compounds for the first time.

Compound **5**, (stavudine d4T), tritylated thymidine nucleoside analogue, eliminated both forms of the parasite without affecting the mammalian cells, as demonstrated by the lack of cytotoxicity against murine peritoneal macrophages up to 50 μM and nonhemolytic activity up to 100 μM. These findings highlighted this hit presented selectivity against *L. infantum* and suggested its in vitro safety profile, like the other chemically related analogues,^27^ indicating a promising biological activity for drug discovery exploration.

Studies of the MoA should be essential early in the drug discovery process. Characterizing the metabolic or signaling pathways perturbed by a candidate can significantly refine therapeutic strategies and facilitate the identification of predictive biomarkers, even in cases where the primary molecular target is not identified.^28^ To investigate the MoA of **5** in *L. infantum*, we performed different experiments using fluorescent-based approaches by spectrofluorometry and fluorescent microscopy. The plasma membrane is fundamental to cellular viability, serving as a selective physical barrier between the extracellular environment and the cytosolic compartment. It facilitates the regulated exchange of solutes, maintains transmembrane electrochemical potentials, and preserves structural integrity. In Trypanosomatids, the plasma membrane is constitutively distinct from that of the mammalian host, rendering it a highly promising drug target.^29^

In our study, we did not observe an alteration in the plasma membrane permeability of *L. infantum* promastigotes treated with **5**. However, the membrane was rapidly depolarized, indicating a disruption of the electrical potential gradient. The difference across the cell membrane is reduced, influencing various processes like ion transport and nutrient uptake.^30^ Similar results with cyclobenzaprine, a muscle relaxant drug, were reported in *L. infantum*.^31^ Nevertheless, depolarization contributed to more severe damage to the parasite; it cannot be singled out as the exclusive cause for the *Leishmania* death.

Unlike mammalian cells, trypanosomatids have a unique mitochondrion with several important metabolic peculiarities, making them an excellent drug target. It has a pivotal role in energy production and participates directly in the establishment of oxidative stress and, consequently, the production of ROS.^32^ In the present study, we did not observe an alteration in the ROS levels of *L. infantum* promastigotes treated with **5**. However, further investigation should be performed in mitochondrial metabolism to determine if direct alteration affects the production of these species, which, in excess, can induce oxidative stress and interfere with metabolic pathways, causing irreversible cellular damage.^32^

In the trypanosomatid, Ca^2+^ plays an important role in cell signaling, which may facilitate their invasion of host cells, respond to environmental changes within the host, or regulate the function of their intracellular organelles.^33^ Intracellular Ca^2+^ homeostasis is maintained through the concerted action of three specialized organelles: the endoplasmic reticulum, the mitochondria, and the acidocalcisomes. Increases in ion concentration can trigger signaling cascades that culminate in parasite death via apoptosis-like mechanisms.^34^ In our studies, we did not observe any alteration in the Ca^2+^ levels in *L. infantum* promastigotes after treatment with **5**, suggesting no disruption of parasite Ca^2+^ homeostasis.

We also investigated the potential participation of the acidocalcisomes, acidic vacuoles containing Ca^2+^, polyphosphates, and other ions. These organelles are the main Ca^2+^ compartment in trypanosomatids, unlike mammalian cells, where the endoplasmic reticulum performs this function.^34^ Acidocalcisomes possess a proton-pumping pyrophosphatase that allows the accumulation of H_3_O, and hence the acidification of these organelles, together with their Ca^2+^ capacity for accumulation. After the alkalinization of this organelle, calcium can be released.^33^ In our studies, no alteration of the acidocalcisomes was observed in *L. infantum* promastigotes treated with **5**. In contrast, the MoA of miltefosine in *L. donovani* is related to a direct effect on the acidocalcisomes and produces a large intracellular Ca^2+^ accumulation.^35^

To assess the impact on genomic integrity, DNA fragmentation analysis was performed on *L. infantum* promastigotes treated with compound **5**. Agarose gel electrophoresis revealed that the compound induced no detectable alterations in genomic DNA integrity. These observations suggest that the antileishmanial activity of compound **5** is not mediated by direct DNA fragmentation, pointing toward an alternative mechanism of action. Further analysis should be performed to elucidate the parasite death pathways, whether it involves necrosis, autophagy, or apoptosis.^36^

However, our findings demonstrate other biological targets that may be exerted by the compound **5**; it did not rule out its involvement in the pyrimidine salvage or de novo pathway. Biochemical and cellular assays should be conducted to identify the specific enzyme targets and validate the pathway involved.^4^

## 4 Conclusions

Our work demonstrates that compound **5** might be a suitable candidate for the development of antileishmanial agents. This analogue, when applied at EC_50_, did not cause damage to plasma membrane permeability, the integrity of the genetic material, acidocalcisomes, intracellular Ca^2+^, and ROS levels of treated *Leishmania* parasites. However, causes a depolarization of the plasma membrane potential, leading to cell death. Further studies are also necessary to understand the enzymatic action of this hit compound, and optimization is required to develop more effective and safer antileishmanial lead compounds.

## 5 Material and Methods

### 5.1 General Experimental

All solvents and reagents were used as obtained from commercial sources. All reactions were performed under an argon atmosphere. The ^1^H and ^13^C NMR spectra were recorded on a Bruker spectrometer (Billerica, MA, USA) operating at 500 MHz for ^1^H and 125 MHz for ^13^C. CDCl_3_ was used as the solvent for NMR experiments, unless otherwise stated. Mass spectra (MS) were measured in positive-mode electrospray ionization (ESI). Thin-layer chromatography (TLC) was performed on silica gel 60 F254 plastic sheets. Column chromatography was performed using silica gel (35–75 mesh) or on an Isolera Biotage system (Uppsala, Sweden). Purity of prepared compounds was determined to be >95% by high-performance liquid chromatography (HPLC)–UV analysis (Thermo HPLC connected with UV detector; Varian Pursuit XS, 4.6 × 150 mm, 5.0 μm).

### 5.2 Chemistry

All tritylated nucleosides were prepared according to the previously reported method.^13^ Briefly, a mixture of a nucleoside (1.0 equiv), an appropriate trityl chloride (2.2 equiv), and dimethylaminopyridine (DMAP, 2.8 equiv) in anhydrous pyridine (4.5 mL/mmol) was heated at 80 °C under argon for 18 h. The reaction was quenched by the addition of MeOH (2 mL/mmol) at rt, and kept stirring at rt for 30 min. The solution was then concentrated and diluted in CH_2_Cl_2_. The organic solution was washed with a saturated solution of NaHCO_3_ (3 × 20 mL), and the combined aqueous layers were extracted with CH_2_Cl_2_. Combined organic layers were dried over Na_2_SO_4_, filtered, and concentrated under vacuum. The residue was purified by column chromatography on silica gel (eluent system gradient MeOH in CH_2_Cl_2_ = 1–3% containing 0.5% triethylamine).

### 5.3 Synthesis of 5-Methyl-1-((2*R*,5*S*)-5-((trityloxy)­methyl)-2,5-dihydrofuran-2-yl)­pyrimidine-2,4­(1*H*,3*H*)-dione (**5**)

Prepared according to the general procedure from 2′,3′-didehydro-2′,3′-dideoxythymidine (d4T) (0.250 g, 1.11 mmol), trityl chloride (0.621 g, 2.23 mmol) in anhydrous pyridine (10 mL). After workup, the crude was purified by column chromatography eluting with dichloromethane/methanol (97%: 3%) to give compound **5** as a white solid (0.415 g, 80%). ^1^H NMR (CDCl_3_, 500 MHz): δ_H_ 8.40 (s, 1H, NH), 7.35–7.29 (m, 7H, H-Ph and H-6), 7.25–7.17 (m, 10H, H-Ph), 7.01–7.00 (m, 1H, H-1′), 6.30 (dt, 1H, *J* = 6.0, 1.5 Hz, CH=), 5.83 (ddd, 1H, *J* = 6.0, 2.0, 1.5 Hz, CH=), 4.93–4.82 (m, 1H, H-4′), 3.40 (dd,1H, *J* = 10.5, 2.5 Hz, H-5′a), 3.37 (dd, 1H, *J* = 10.5, 4.0 Hz, H-5′b), 1.97 (d, *J* = 1.5 Hz, 3H, CH_3_); ^13^C (CDCl_3_, 125 MHz): δ_c_ 162.6 (CO), 149.6, (CO), 142.1, 135.0­(C–H_thy)_, 133.8 (CH=), 127.7­(C_ArH_), 126.9 (C_ArH_), 126.4 (C_ArH_), 125.4 (CH=), 110.2­(CMe), 88.9 (C-1′), 85.9, 84.7 (C-4′), 63.8 (C-5′), 28.7 (Ctrit), 10.3 (CH_3_). MS (ES+), found: *m*/*z* 489.19 (M+ Na+); Calculated for [C_29_H_26_N_2_O_4_]: *m*/*z* 466.1893 (M); Reverse-phase HPLC (H_2_O/CH_3_CN from 80/20 to 0/100 in 30 min), flow = 1 mL/min, λ = 265 nm, *t*_R_ = 20.35 min (see ^13^C NMR spectra in Supporting Information, S1 and S2).

### 5.4 Synthesis of 2-(Tritylamino)-9-(4-(trityloxy)-3-((trityloxy)­methyl)­butyl)-1,9-dihydro-6*H*-purin-6-one (**9**)

Prepared according to the general procedure above. White solid (0.47 g, 21%); ^1^H NMR (500 MHz, 25 8C, d_6_-DMSO): δ_H_ = 10.51 (bs, 1 H, NH), 7.26 (s, 1 H, H-8), 7.31–7.28 (m, 31 H, H-Ph, NH), 7.19–7.17 (m, 6 H, H-Ph), 7.07–7.04 (m, 6 H, H-Ph), 6.97–6.96 (m, 3 H, H-Ph), 3.24–3.21 (m, 2 H, H-1′), 2.91–2.85 (m, 4 H, H-4′, H-5′), 1.62–1.58 (m, 1 H, H-3′), 1.13–1.09 ppm (m, 2 H, H-2′); ^13^C NMR (125 MHz, d_6_-DMSO): δ_c_ = 156.5 (C-6), 150.3 (C-2), 149.3 (C-4), 144.6, 143.8 (‘ipso’ C-Ph), 137.2 (C-8), 128.7, 128.4, 128.1, 127.8, 127.4, 126.9, 126.3 (CH-Ph), 117.0 (C-5), 85.8 (C­(Ph)_3_), 62.7 (C-4′, C-5′), 41.3 (C-1′), 37.3 (C-3′), 28.4 ppm (C-2′); MS (ES+) found: *m*/*z* 1003.39 [M + Na]+, calculated for [C_67_H_57_N_5_O_3_] *m*/*z*: 980.20 [M]; Reverse phase HPLC (H_2_O/CH_3_CN from 50:50 to 0:100 in 40 min), flow = 1 mL/min, l = 254 nm, *t*_R_ = 36.64 min.

### 5.5 Synthesis of 2-Amino-9-(4-(trityloxy)-3-((trityloxy)­methyl)­butyl)-1,9-dihydro-6*H*-purin-6-one (**10**)

Prepared via our general procedure as a white solid (0.15 g, 9%). ^1^H NMR (d_6_-DMSO-d6, 500 MHz) δ_H_ = 10.56 (bs, 1H, NH), 7.39 (s, 1H, H-8), 7.33–7.24 (m,30H, H-Ph), 6.37 (bs, 2H, NH_2_), 3.67 (t, *J* = 6.9 Hz, 2H, H-1′), 3.15–3.12 (m, 2H, H-4′), 3.08–3.05 (m, 2H, H-5′), 1.79–1.78 (m, 1H, H-3′), 1.74–1.70 (m, 2H, H-2′); ^13^C NMR (d_6_-DMSO, 125 MHz): δ_C_ = 156.8 (C-6), 153.37 (C-2), 151.0 (C-4), 143.8 (‘ipso’ C-Ph), 137.0 (C-8), 128.5, 128.17, 127.8, 127.5, 126.9 (CH-Ph), 116.7 (C-5), 85.8 (C­(Ph)_3_), 62.6 (C-4′, C-5′), 40.9 (C-1′), 36.9 (C-3′), 28.4 (C-2′). MS (ES+) Found: *m*/*z* 760.43 (M + Na+); Calculated for [C_48_H_43_N_5_O_3_]: *m*/*z* 737.89 (M); Reverse-phase HPLC (H_2_O/CH_3_CN from 50/50 to 0/100 in 30 min), flow = 1 mL/min, λ = 254 nm, *t*_R_ = 18.64 min.

### 5.6 Animal, Parasites, and Mammalian Cell Maintenance

*Leishmania (Leishmania) infantum* (MHOM/BR/1972/LD) was maintained in golden hamsters (*Mesocricetus auratus*) for up to 60–70 days postinfection. The amastigote forms were obtained from the spleen of previously infected hamsters and purified by differential centrifugation.

*L. infantum* promastigotes forms were cultured in 199 medium (M199) supplemented with 10% inactive fetal bovine serum (FBS), 0.25% hemin, and 5% human urine at pH 7.2 in a B.O.D. incubator at 25 °C.

Peritoneal macrophage cells collected from the peritoneal cavity of BALB/c mice (*Mus musculus*) by washing with RPMI-1640 medium supplemented with 10% FBS and murine conjunctive fibroblast cells (NCTC clone 929, ATCC) were maintained in RPMI-1640 supplemented with 10% FBS at pH 7.2 and 37 °C in a humidified incubator with 5% CO_2_.

Animal procedures were performed with the approval of the Research Ethics Commission of Adolfo Lutz Institute/SP/Brazil (project CEUA/IAL 05/2021) in agreement with the Guide for the Care and Use of Laboratory Animals from the National Academy of Sciences (http://www.nas.edu).

### 5.7 Phenotypic Screening Assay against *L. infantum*

Extracellular promastigotes (late growth phase, 1 × 10^6^/well) were added to 96-well plates in the presence of the compounds, serially diluted (150–0.58 μM) in M199 + 10% FBS and incubated for 48 h in a B.O.D. incubator at 25 °C. Parasite viability was determined using the MTT colorimetric method. Untreated cells were used as a negative control (100% viability) and miltefosine as a standard drug.

Activity against intracellular *L. infantum* amastigotes was determined in infected macrophages. Macrophages were collected from the peritoneal cavity of BALB/c mice by washing with RPMI-1640 + 10% FBS and seeded at 1 × 10^5^ cells/well in 16-well slide chambers for 24 h. Amastigotes were isolated from the spleens of previously infected hamsters, purified by differential centrifugation, and added to the macrophages (1 macrophage: 10 amastigotes) for 24 h at 37 °C in 5% CO_2_. Noninternalized parasites were removed by washing once with medium, and the cells were then incubated with the compounds serially diluted (6.25–50 μM) for 96 h at 37 °C. The cells were fixed in methanol, stained with Giemsa, and observed under a light microscope. The number of amastigotes was determined in a total of 200 macrophages from the treated and untreated cells. Untreated cells were used as a negative control (100% infectivity), and miltefosine was used as a standard drug. The infection index (II) was determined using the following equation: II = (number of infected macrophages x number of amastigotes)/total macrophages.^37^

### 5.8 2D Cell Cytotoxicity Phenotypic Assay

NCTC cells (6 × 10^4^/well) or peritoneal macrophages from BALB/c mice (6 × 10^4^/well) were added to 96-well plates in the presence of the compounds, serially diluted (200–1.56 μM) in RPMI-1640 + 10% FBS and incubated for 48 h in a humidified incubator with 5% CO_2_. Cell viability was determined using the MTT colorimetric method. Untreated cells were used as a negative control (100% viability) and miltefosine as a standard drug. The selectivity index (SI) was calculated by the ratio: CC_50_ against NCTC cells/EC_50_ against amastigotes.^37^

### 5.9 Hemolytic Activity

Erythrocytes were collected from BALB/c mice. In brief, erythrocyte suspension with 3% (v/v) HBSS was treated with compound **5** serially diluted (200–1.56 μM) in HBSS and incubated for 2 h at 37 °C. Samples were centrifuged, and the supernatant was taken for absorbance at 570 nm. Bidistilled water was employed as a positive control, and untreated erythrocytes as a negative control.^38^

### 5.10 Evaluation of the Mechanism of Action in *L. infantum*

The most active compound, in both forms of the parasite, was subjected to the mechanism of action (MoA) analysis. Previously, a new EC_50_ assay was performed in promastigotes in short-term incubation. Parasites (2 × 10^6^/well) were treated with compound **5** serially diluted (150–0.58 μM) for 1, 2, and 4 h, at 25 °C. Parasite viability was determined using the MTT colorimetric method. Untreated cells were used as a negative control.

#### 5.10.1 Plasma Membrane Integrity Assays

##### 5.10.1.1 Plasma Membrane Permeability

Promastigotes (late growth phase, 2 × 10^6^/well) were washed in HBSS and incubated in a 96-well black microplate with 1 μM SYTOX Green fluorescent probe (Molecular Probes) for 15 min at 25 °C. Then, **5** was added at the EC_50_ (55 μM), and the fluorescence was measured every 10 min up to 120 min using a fluorimetric microplate reader (Filter Max F5Multi-Mode Microplate Reader, Molecular Devices) with excitation and emission wavelengths of 485 and 520 nm, respectively. Untreated cells were used as a negative control, and cells treated with 0.5% (v/v) Triton X-100 were used as a positive control.^39^

Promastigotes (late growth phase, 1 × 10^6^/well) were incubated with the **5** at the EC_50_ (55 μM) for 2 h at 25 °C. Cells were washed with PBS, and a pool of 2 × 10^7^ was incubated with 1 μM SYTOX Green for 15 min at 25 °C. Then, cells were washed with PBS, resuspended in glycerol phosphate buffer, and 20 μL was added to a glass slide. The cells were observed under a digital fluorescence microscope (EVOS M5000 Imaging System, Thermo Fisher Scientific). Untreated cells were used as a negative control, and cells treated with 0.5% (v/v) Triton X-100 were used as a positive control.

##### 5.10.1.2 Plasma Membrane Electric Potential

Promastigotes (late growth phase, 2 × 10^6^/well) were washed in HBSS and incubated in a 96-well black microplate with 0.2 μM DiSBAC_2_(3) fluorescent probe (Molecular Probes) for 5 min at 25 °C. Then, **5** was added at the EC_50_ (55 μM), and the fluorescence was measured every 10 min up to 120 min using a fluorimetric microplate reader, with excitation and emission wavelengths of 544 and 584 nm, respectively. Untreated cells were used as a negative control, and cells treated with 35 μM of miltefosine were used as a positive control.^31^

Promastigotes (late growth phase, 1 × 10^6^/well) were incubated with the **5** at the EC_50_ (55 μM) for 2 h at 25 °C. Cells were washed with PBS, and a pool of 2 × 10^7^ was incubated with 0.2 μM DiSBAC_2_(3) probe for 5 min at 25 °C. Then, cells were washed with PBS, resuspended in glycerol phosphate buffer, and 20 μL was added to a glass slide. The cells were observed under a fluorescence microscope (Olympus BX 60, Olympus Life Science). Untreated cells were used as a negative control, and cells treated with 35 μM of miltefosine were used as a positive control.

#### 5.10.2 Genomic DNA Integrity Assays

##### 5.10.2.1 Agarose Gel Electrophoresis

Promastigotes (late growth phase, 1 × 10^6^/well) were incubated with the **5** at the EC_50_ (55 μM) for 2 h at 25 °C. Cells were washed with PBS, and a pool of 2 × 10^7^ was subjected to DNA extraction with the QIAamp DNA Mini Kit (Qiagen), according to the manufacturer’s instructions. Purity and quantity were determined by spectrophotometry (Nanodrop ND100, Thermo Fischer Scientific). The DNA samples were analyzed in a 2% agarose gel stained with ethidium bromide, and electrophoresis was performed for 40 min at 400 mA and 100 V. The result was visualized using a UV transilluminator MiniBIS Pro (DNA Bioimaging System). Untreated cells were used as a negative control, and cells treated with 4 mM of hydrogen peroxide were used as a positive control.^39^

##### 5.10.2.2 Cellular Electrophoresis in Microgel (Comet Assay)

Promastigotes (late growth phase, 1 × 10^6^/well) were incubated with the **5** at the EC_50_ (55 μM) for 2 h at 25 °C. Cells were washed with PBS and resuspended in 200 μL, subjected to a pool of 2 × 10.^7^ Then, 5 μL of the cell pellet was added to 115 μL of low-melting-point agarose (0.75%), and 50 μL was distributed in pregelled slides with normal-melting-point agarose. After cooling for 5 min, slides were immersed in a lysis solution (2.5 M NaCl, 100 mM EDTA, 10 mM Tris) and were refrigerated at 4 °C for 1 h. Slides were incubated for 20 min in an alkaline buffer (10 M NaOH, 0.2 M EDTA, and distilled water [pH 13]), followed by electrophoresis at 25 V and 300 mA for 25 min. Slides were neutralized (0.5 M Tris-HCl [pH 7.5]) for 15 min, dried at room temperature, and fixed in 100% ethanol. They were stained with ethidium bromide (20 μg/mL) and were analyzed under a fluorescence microscope (Olympus BX60, Olympus Life Science, 516-to-560 nm filter; 590 nm barrier filter; 40 lens).^40^

#### 5.10.3 Oxidative Metabolism Assays

##### 5.10.3.1 Reactive Oxygen Species (ROS)

Promastigotes (late growth phase, 2 × 10^6^/well) were washed in HBSS and incubated in a 96-well black microplate with 5 μM H_2_DCFDA fluorescent probe (Molecular Probes) for 15 min at 25 °C. Then, **5** was added at the EC_50_ (55 μM), and the fluorescence was measured every 10 min up to 120 min using a fluorimetric microplate reader, with excitation and emission wavelengths of 485 and 520 nm, respectively. Untreated cells were used as a negative control, and cells treated with 10 μM of sodium azide were used as a positive control.^37^

Promastigotes (late growth phase, 1 × 10^6^/well) were incubated with the **5** at the EC_50_ (55 μM) for 2 h at 25 °C. Cells were washed with PBS, and a pool of 2 × 10^7^ was incubated with a 5 μM H_2_DCFDA fluorescent probe for 15 min at 25 °C. Then, cells were washed with PBS, resuspended in glycerol phosphate buffer, and 20 μL was added to a glass slide. The cells were observed under a fluorescence microscope (Olympus BX 60). Untreated cells were used as a negative control, and cells treated with 10 μM of sodium azide were used as a positive control.

##### 5.10.3.2 Intracellular Calcium Ions (Ca^2+^)

Promastigotes (late growth phase, 2 × 10^6^/well) were washed in HBSS and incubated with Fluo-4 AM (5 μM) for 1 h at 25 °C. Cells were washed in HBSS and maintained for 30 min at 25 °C. Then, **5** was added at the EC_50_ (55 μM), and the fluorescence was measured every 20 min up to 120 min using a fluorimetric microplate reader, with excitation and emission wavelengths of 485 and 535 nm, respectively. Untreated cells were used as a negative control, and cells treated with 0.5% (v/v) Triton X-100 were used as a positive control.^38^

Promastigotes (late growth-phase, 1 × 10^6^/well) were incubated with Fluo-4 AM (5 μM) for 1 h at 25 °C. Cells were washed in HBSS and maintained for 30 min at 25 °C. Then, **5** was added at the EC_50_ (55 μM) for 2 h at 25 °C. Cells were washed with PBS, a pool of 2 × 10^7^ was resuspended in glycerol phosphate buffer, and 20 μL was added to a glass slide. The cells were observed under a digital fluorescence microscope (EVOS M5000 Imaging System). Untreated cells were used as a negative control, and cells treated with 0.5% (v/v) Triton X-100, as a positive control.^41^

##### 5.10.3.3 Acidocalcisomes

Promastigotes (late growth phase, 2 × 10^6^/well) were washed in HBSS and incubated with acridine orange (4 μM) for 5 min at 25 °C. Cells were washed in HBSS, and **5** was added at the EC_50_ (55 μM). Fluorescence was measured every 20 min up to 120 min using a fluorimetric microplate reader, with excitation and emission wavelengths of 485 and 535 nm, respectively. Untreated cells were used as a negative control, and cells treated with nigericin (4 μM) were used as a positive control.^38^

Promastigotes (late growth phase, 1 × 10^6^/well) were incubated with acridine orange (4 μM) for 5 min at 25 °C. Cells were washed, and **5** was added at the EC_50_ (55 μM) for 2 h at 25 °C. Cells were washed with PBS, a pool of 2 × 10^7^ was resuspended in glycerol phosphate buffer, and 20 μL was added to a glass slide. The cells were observed under a digital fluorescence microscope (EVOS M5000 Imaging System). Untreated cells were used as a negative control, and cells treated with nigericin (4 μM) were used as a positive control.^41^

### 5.11 Statistical Analysis

The Effective Concentration 50% (EC_50_) and Cytotoxic Concentration 50% (CC_50_) data represent the mean and the standard error of the mean of three representative independent assays, in which the sample was tested in duplicate. They were calculated using the dose–response sigmoid curves generated in GraphPad Prism 5.0 (GraphPad Software, San Diego, CA, USA) with analysis of the respective 95% confidence intervals and linear coefficients (*r* ^2^). All data from MoA represent the mean and the standard deviation of two representative independent assays, which sample tested in triplicate. The data were subjected to one-way analysis of variance ANOVA, applying Tukey’s multiple comparison test, with *p* < 0.05 considered to indicate a statistically significant difference.

## Supplementary Material



## Notes

^13^C NMR spectra of compound **5** (PDF)
