# Supplementary material for: Nucleoside Analogue with Thymidine Nucleobase Inhibits Leishmania infantum and Depolarizes the Plasma Membrane Potential In Vitro
Source: ACS Omega. 2026 Feb 26;11(9):14414–25. doi: 10.1021/acsomega.5c09199 (PMC12980188; doi:10.1021/acsomega.5c09199)
Supplement: Supplementary file 1 [file ao5c09199_si_001.pdf]

Nucleoside analogue with thymidine nucleobase inhibits *Leishmania infantum* and depolarizes the plasma membrane potential in vitro

Clarissa Menezes[1;2]; Ingrid de O. Dias[1;2]; Elisa Pileggi[3], Andre G. Tempone[4]; Fabrizio Pertusati[3,*]; Samanta E. T. Borborema[1,*]

[1]Center for Parasitology and Mycology, Adolfo Lutz Institute, Sao Paulo, 01246-000, Brazil

[2]Post-Graduation Program of the Diseases Control Coordination, Secretary of State of Health of Sao Paulo, Sao Paulo, 01246-900, Brazil

[3]School of Chemistry, Cardiff University, Cardiff, CF10 3AT, Wales, United Kingdom

[4]Physiopathology Laboratory, Butantan Institute, Sao Paulo, 05503-900, Brazil

*Corresponding authors: Instituto Adolfo Lutz, Av Dr Arnaldo, 351, 8 andar, 01246-000, Sao Paulo, Sao Paulo, Brazil. samanta.borborema@ial.sp.gov.br; samantaborborema@gmail.com (S.E.T. Borborema); Cardiff University, Main Building, Floor 1, Room 1.89C, Park Place, Cardiff, CF10 3AT, Wales, United Kingdom; pertusatiF1@cardiff.ac.uk (F. Pertusati)

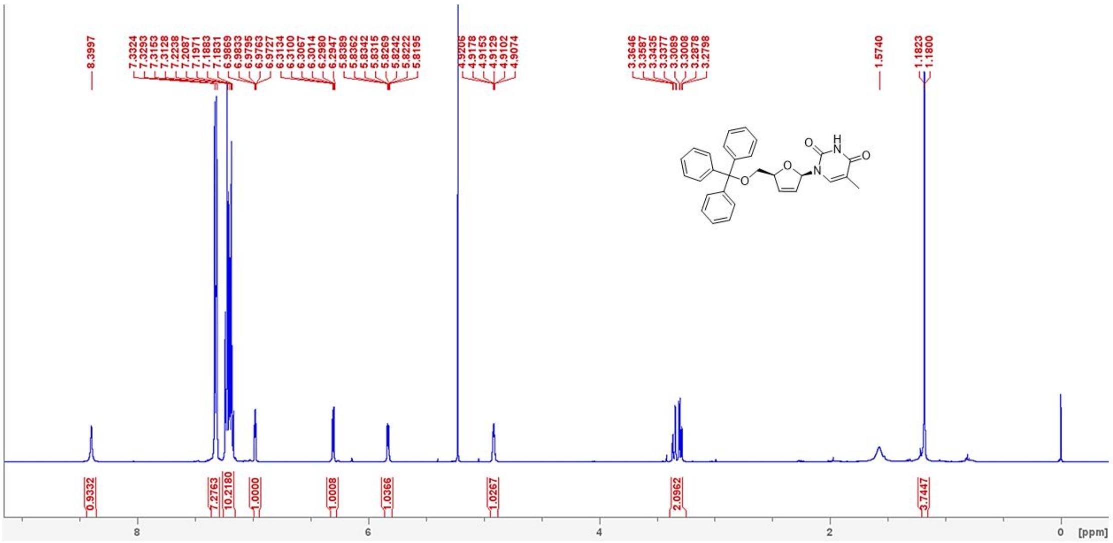

Figure S1. ^{13}C NMR spectra of compound **5** (125 MHz, $CDCl_3$)

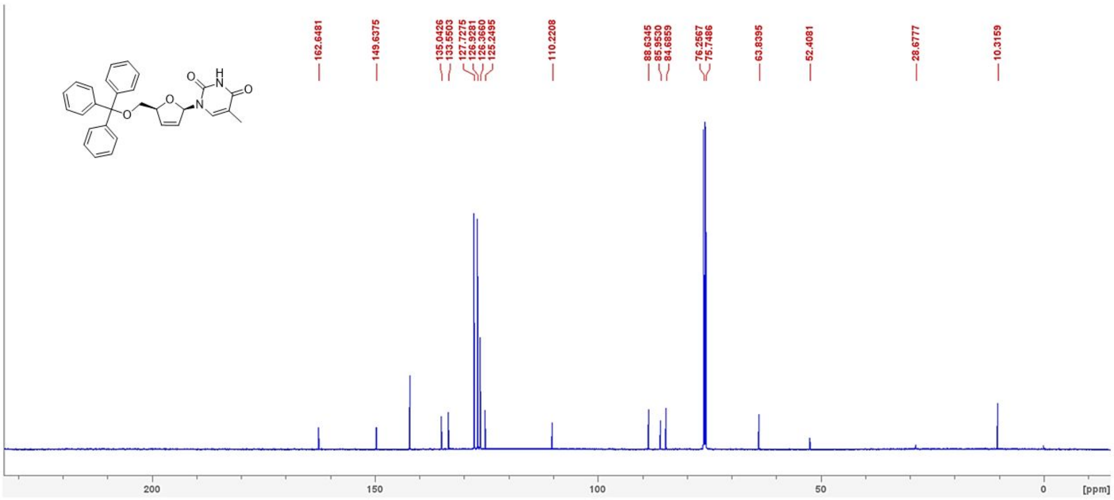

Figure S2. ^{13}C NMR spectra of compound **5** (125 MHz, $CDCl_3$)
